# Progress toward Medical Unity

**Published:** 1903-06

**Authors:** 


A Monthly Review of Medicine and Surgery.

*EDITOR:*

WILLIAM WARREN POTTER, M. D.

All communications, whether of a literary or business nature, books for review and exchanges should be addressed to the editor: 284 Franklin Street, Buffalo, N. Y.

Vol. XLII.—LVIII. JUNE, 1903. No. 11.

### Progress Toward Medical Unity.

THE American Medical Association during its session at New Orleans, May 5-8, 1903, took the most progressive step in its history. The Code of Ethics adopted as an admonitory document in 1847, made mandatory by the surreptitious insertion of a clause in the constitution in 1857, enforced as a blue-law with disastrous results to the profession until 1901, eliminated partially in that year and completely in 1902 from the organic law of the Association, has at last been given a new name, a new form, a new and, we believe, an entirely satisfactory status.

It is unnecessary to review the events leading up to this result. The unfortunate misunderstanding at St. Paul twenty and more years ago; the division of the medical profession in the state of New York; the adoption by the American Medical Association of resolutions explanatory of the code; the enactment of salutary medical laws by the different states; the rapid advances in medical education; the reorganisation of the American Medical Association; the organisation on distinctly non-sectarian lines of societies in affiliation with it; the elimination of the Code of Ethics from the organic law of the national association; the appointment of a committee to revise the eliminated code; conferences for the reunion of the profession in New York State; the report of the committee on revision of the eliminated code; the submission of a substitute set of "Principles"; the appointment of conference committees; the submission of the final report and its unanimous adoption, make one of the most interesting and important chapters in the history of American medicine. History, however, is of yesterday; we are interested, today, only in the precise results of the New Orleans meeting. What has been done?

The answer should relate first to the Code itself. The changes in this document were many, those of major importance being the following:

First.—The title "Code of Ethics" was dropped and that of "Principles of Ethics" was substituted, the house of delegates thereby relieving the document of even a verbal resemblance to a statutory enactment.

Second.—A preamble was adopted, stating explicitly that "the American Medical Association promulgates as a suggestive and advisory document" the statement of principles that followed. This preamble was adopted separately with the distinct declaration by vote that it was to be the preamble of any statement of ethical principles which might subsequently be agreed upon. The house of delegates thus specifically determined not only that the Code was finally and definitely out of the organic law, but that it was out to stay out. This point having been definitely and emphatically determined the exact phraseology of the principles subsequently to be adopted became a matter of relatively little importance.

Third.—The restrictive clause relating to consultations was entirely eliminated—an act by which the long standing and offensive barrier to individual liberty in the matter of professional associations is finally and definitely removed.

Fourth.—The importance of medical organisation was emphasised while no attempt was made to define who were or were not eligible to membership in subordinate and affiliated societies, except that they must be practitioners of medicine under the law. This action by the house of delegates was really made necessary to give ethical sanction to the active work of organisation that its official representatives are stimulating and effecting in several states.

Fifth.—The adjustment of ethical questions was relegated to the state associations and to their constituent societies, which are invested with the largest discretionary power in such matters.

Sixth.—All suggestions for the guidance of the public in its relations to the profession were stricken out.

So much for the "Code" itself—or rather for the "Principles" as the new document must now be designated. There were, however, other results of equally striking importance.

The long and bitter controversy that was expected did not come off. The report of the committee was presented and Dr. Reed offered a substitute for the Code that the committee had published as its report. The house of delegates very promptly adopted the preamble presented in the substitute whereby any document that might later be agreed upon would be purely advisory. Thereafter agreement became easy. The conference was held without serious differences arising and with the result that additional changes were made. When, finally, the completed document reached the house of delegates it was adopted with manifestations of enthusiasm rarely seen in a deliberative body. There were not only sighs of relief, there were shouts of joy, that the troublesome question was out of the way.

The action was distinctly expressive of the sentiment of the medical profession of the country. Local conditions in the state of New York were not taken into consideration. There was no question of expediency. What was done by the house of delegates was manifestly that which in its judgment was best for the profession, without reference to incidental or extraneous considerations. The final result, it seems to us, could not have been better devised to solve the problems awaiting adjustment in the state of New York, as well as the problems of medical organisation of the entire country.

---

THE House of Delegates did unusually good legislative work and by its industry and practical application demonstrated the wisdom of its creation. Dr. Frank Billings made an admirable presiding officer. His dignity of bearing and good judgment guided the house through eventful work without friction or disturbance. The president-elect, Dr. Musser, of Philadelphia, not being present could not be installed, so under the rules the house directed Dr. Billings to continue in office until the session of 1904 convenes. It is not probable that hereafter the election of an absentee will happen.

---

THE changed attitude of the association regarding the code of ethics is due to the persistent efforts of Dr. Charles A. L. Reed, of Cincinnati, who some time ago determined, if within his power to prevent, that no obsolete rules of personal conduct should longer be carried on the statute books, particularly as nobody pretended to live up to them. Dr. Reed's substitute introduced at New Orleans gave him the lever-hand in the race, and made it impossible for any action to be taken that did not receive the approval of him and his inclining.

---

It is very important that only experienced men be sent to the house of delegates. Those unfamiliar with the rules are prone to delay action or provoke unwise legislation. It is a good plan to return the efficient men for at least a few terms, or until the rules and precedents become sufficiently established to ensure harmonious working of legislative methods.

---

The registered attendance at the meeting was stated at a fraction over 2,000, which has become the accepted average within the last few years. This, of course, means that at least 2,500 visitors were present. The arrangements were admirable and all were well pleased, thanks to the efficiency of the committee with Dr. Isadore Dyer at its head.

---

The medical editors' dinner at Antoine's, Monday evening, was unusually well ordered as to decorations, menu and service. A vocal quartet of negroes gave a novelty to the entertainment and added enthusiasm to the occasion. The president, Dr. Winslow Anderson, of San Francisco, in announcing the toasts stated that in each instance no previous notice had been given the speakers, but, even so, the responses were spirited and in many instances eloquent.

